# Looking into the Content of the Canadian Occupational Performance Measure (COPM): A Danish Cross-Sectional Study

**DOI:** 10.1155/2020/9573950

**Published:** 2020-05-14

**Authors:** Anette Enemark Larsen, Sonja Wehberg, Jeanette Reffstrup Christensen

**Affiliations:** ^1^Occupational Therapy, Department of Therapist and Midwifery, The Faculty of Health Sciences, Copenhagen University College, Copenhagen, Denmark; ^2^Research Unit for General Practice, Department of Public Health, University of Southern Denmark, Denmark

## Abstract

**Purpose:**

To examine the content validity of the Danish version of the Canadian Occupational Performance Measure (COPM-DK).

**Materials and Methods:**

This cross-sectional study was performed in a hospital and a community rehabilitation centre. The content validity of the COPM was assessed by relating the clients' prioritized occupational performance issues (OPIs) to the conceptual model of the Canadian Model of Occupational Performance and Engagement (CMOP-E) and the levels of the Taxonomic Code of Occupational Performance (TCOP). Six occupational therapy lecturers participated in classifying the OPIs using the TCOP.

**Results:**

A total of 112 clients from a regional and community-based rehabilitation participated. The 56% regional participants came from a hospital's hand and knee surgery department. The remaining 44% participants came from a community-based rehabilitation centre with in- and outpatient departments. There were 44% males, with a mean age of 65.2 years. They prioritized 495 OPIs, of which 40% concerned self-care, 32% productivity, and 28% leisure. The prioritized OPIs were divided into a total of 224 different OPIs. There were significant differences in which areas were prioritized in the various population groups. Of the OPIs, 64.3% could be classified into the TCOP levels of occupation and activity, i.e., 1/3 of the OPIs were related to tasks and actions, and thus beyond the scope of the COPM. The interrater agreement of the OPI classification was only fair (kappa 0.3).

**Conclusion:**

The content validity of the COPM seems to depend on how and with which clients it is administered. Caution must be taken to secure OPIs on the higher levels of the TCOP, while maintaining the clients' right to nominate OPI preferences. Therefore, an introductory course and on-going support are recommendable. Bearing this in mind, the COPM seems useful to identify individual clients' prioritized OPIs in a Danish context.

## 1. Introduction

The Canadian Occupational Performance Measure (COPM) is a client-centred assessment meant to help clients identify, prioritize, and evaluate issues in the important occupations they encounter in their lives [1]. Being that the COPM helps clients identify their important occupations, it facilitates a client-centred approach, including partnership and collaboration between the professional and the client in the forthcoming rehabilitation [2–5]. Concentrating on issues of importance to the clients is an integrated part of an occupational therapy (OT) intervention [4, 6] as it enhances the effect of the rehabilitation and the clients' perceived satisfaction with the rehabilitation [7, 8]. The COPM is thus a valued tool at the outset of rehabilitation [9, 10]. The COPM is widely used by occupational therapists (OTs) and other health professionals [11, 12], with a track record of more than 25 years and is available in 36 languages [1]. A substantial amount of research documents its good clinimetric properties, and it works well as an assessment and outcome measure in clinical practice [13–15].

According to the manual, the COPM is based on an explicit model of OT, the Canadian Model of Occupational Performance and Engagement (CMOP-E), in which enablement of occupation and a client-centred approach are central [1]. The COPM encompasses the occupational performance areas of the CMOP-E as primary outcomes, and in the CMOP-E, occupational performance is defined as “…the ability to choose and satisfactorily perform meaningful occupations that are culturally defined and age appropriate*…*” (16, p. 30). It is depicted as the interaction between a person (having certain performance capacities and a spiritual self), interacting with occupations (divided into three areas), and in the environment (defined by the cultural, social, physical, and institutional surroundings) [16]. The three occupational performance areas of the CMOP-E are *self-care* (S), what a person needs to do (i.e., personal care and basic needs); *productivity* (P), what a person is obliged to do (i.e., contribution to the social and economic fabric in the community), and *leisure* (L), what a person wants to do (i.e., how to enjoy life) [1, 16]. Furthermore, in the CMOP-E, the authors have made a distinction between occupational performance and occupational engagement, where the concept of occupational performance has been broadened to encompass the overall conception of the human need for having occupations and not only performing them, i.e., to include the concept of engagement [16].

Research has shown that for the COPM to be valid users have to understand the concept of occupation and occupational performance as understood by the CMOP-E [5, 11, 15]. From the early days of the OT profession, many scholars have attempted to define and conceptualize the terms of occupation and occupational performance [17–20]. For instance, in 1987 Evans reflected on “Occupation as the core concept of OT,” with occupations being described as the top of a hierarchy, going from the human organism of body systems to human interaction with the external world, i.e., from basic physiological survival to a sense of belonging and human self-actualization [21]. In 2004, a corresponding need to remedy a lack of consistency in the vocabulary of OT was recognized by Polatajko and colleagues, which lead to the proposal of a taxonomy, the Taxonomic Code of Occupational Performance (TCOP) [22]. Alongside the CMOP-E, the TCOP was further developed and presented with five levels, having “occupation” at the top, defined as “an activity or set of activities that is performed with some consistency and regularity, that brings structure, and is given value and meaning by individuals and a culture” (17, p. 19).

Given that the COPM was developed in Canada, the original versions are published in English and French [1]. Therefore, a translation and cross-cultural adaptation process are necessary to assure the measurement's content validity, as well as uniformity of administration and interpretation across languages and cultures [23, 24]. As this had not been undertaken in the first Danish version of the COPM from 2000, the Danish OT Association initiated a cross-cultural translation process with the release of the 5^th^ version of the COPM manual in 2015 [1]. The new Danish version of the COPM, the COPM-DK was released by the end of 2015 [25, 26]. Since content validity is a prerequisite for other validity studies [27], a critical step in the translation process was to examine the content validity, as it addresses several aspects of the measurement's quality (construct definition, adequacy, clarity, and relevance) [28, 29]. Content validity can provide information regarding typicality and clarity of items and help improve measurements through the recommendations of a panel of experts [27]. Thus, in the translation process into Danish, the aspect of content validity was given special attention and examined as recommended by the content validity index (CVI) and debriefing interviews [30, 31]. The study revealed issues with the content validity of the COPM-DK [25]. For instance, it appeared that the participating OTs' work settings and their level of experience and need for guidance from the manual influenced how they administered the COPM interview with regard to their focus on the levels and areas of interest of the identified occupational performance issues (OPIs) [25].

Since content validity is defined as “the degree to which the content of a measurement instrument is an adequate reflection of the construct to be measured” (32, p. 154), data obtained with the COPM should reflect occupational performance, i.e., the content of the CMOP-E, and thus represent the top level of the TCOP. Because, previous studies have highlighted the uncertainties about the content validity of the COPM [5, 25], this study is aimed at examining the content validity of the COPM-DK based on the following research questions:

1. How do the OPIs identified by Danish clients reflect the construct of occupational performance as conceptualized by the CMOP-E
2. How do the identified OPIs relate to the levels of the TCOP

## 2. Materials and Methods

### 2.1. Design and Setting

The study had a quantitative single group pretest design and lasted from February to August 2016. Upon being referred to rehabilitation in two settings in the Capital Region of Denmark, participants were consecutively recruited. The first setting (A) was a regional hospital, where four experienced OTs (work experience between 3 and 18 years) were recruited to assess participants from a hand and a knee surgery department, i.e., the populations referred to as AH and AK. The second setting (R) was a rehabilitation centre, where four experienced OTs (work experience between 5 and 20 years) were recruited to assess participants from in- and outpatient departments, i.e., the populations referred to as RI and RO. All the participating OTs had used the COPM regularly as part of their work experience. Furthermore, the first and the last author and four experienced lecturers from OT departments in Danish Universities and University Colleges participated in the analyses of how the OPIs related to the levels of the TCOP.

### 2.2. Procedure

After being referred to rehabilitation, all newcomer clients were asked to participate in the present research and received verbal and written information about the study. Participants who consented to partake submitted an informed and written consent as part of the process [32]. No later than three working days after consentience, the COPM-DK [25, 26] was administered either by the first author or by one of the participating OTs from the two settings.

After completion of the COPM interviews, the first author manually coded the OPIs. First, all the different OPIs were listed in an excel sheet, in the three occupational performance areas defined by the COPM manual, and given a consecutive number [26]. Then, the five levels of TCOP were each given a colour, and the first and the last author, together with the four lecturers, coloured each OPI according to which level they perceived it belonged to. Finally, the first author manually listed all the replies into one sheet, permuting colour coding into numbers.

### 2.3. Participants

The participants were all Danish-speaking adults in need of rehabilitation due to physically induced conditions like hand injuries or knee replacements (populations AH and AK) or due to symptoms raised from orthopaedic, medical, or neurological diseases and/or injuries (populations RI and RO). We included all newcomer clients consecutively if they needed rehabilitation or a hip replacement and if their communicative and cognitive abilities enabled their participation in a COPM interview. The study complied with the prescribed ethical principles [32] and was registered by the Copenhagen University College with id no. 18-025.

### 2.4. Measurements

#### 2.4.1. The COPM-DK

The COPM is a semistructured interview in five steps. First, in a narrative manner, the OTs helped the participants identify their perceived OPIs within three occupational areas defined in the CMOP-E [16]. According to the COPM manual, the three areas are subdivided as follows: self-care (S) is divided into personal care, functional mobility, and community management; productivity (P) is divided into paid or unpaid work, household management, and school and play; leisure (L) is divided into quiet recreation, active recreation, and socialization (1, p. 3).

Having identified all relevant OPIs, the participants scored the *importance* of the OPIs (the COPM-I), followed by choosing a maximum of five OPIs to be the focus of the forthcoming rehabilitation. The participants scored the chosen OPIs concerning how they perceived their *performance* of the prioritized OPIs (the COPM-P) and how *satisfied* they were with that performance (the COPM-S). The three scores COPM-I, COPM-P, and COPM-S are all VAS scores going from “1,” having the lowest importance, performance, or satisfaction to “10,” having the highest importance, performance, or satisfaction [1, 26]. According to the COPM manual, the COPM-P and COPM-S can be repeated to enable a calculation of the difference between the first and final scores. In order to evaluate our research questions, we only needed data on which OPIs the participants identified and how they scored the COPM-I. Therefore, only the first three steps of the COPM were included.

#### 2.4.2. The TCOP

The TCOP introduces a taxonomy code to differentiate the five levels included in occupations [16]. As described in Table 1, the top level is occupation, followed by activity, task, action, and body function.

### 2.5. Analytic Procedures and Statistics

We made a descriptive and manually based coding and analysis of the prioritized OPIs. First, the codes were divided into the three occupational areas: S, P and L. In the COPM manual, each area was further subdivided into three **(**1**)**, which we labelled A, B, and C. This led to the following nine occupational areas: In self-care, the codes were personal care (SA), functional mobility (SB), and community management (SC). In productivity, the codes were paid or unpaid work (PA), household management (PB), and school and play (PC). In leisure, the codes were quiet recreation (LA), active recreation (LB), and socialization (LC). Based on the OPI codes, descriptive statistics were made to find frequencies, including percentages of the number of OPIs for each of the nine occupational areas, within each population group, each setting, and the entire study population. We tested for homogeneity of the three main occupational areas across population groups with a chi-square (*χ*^2^) test. The frequencies of the OPI codes were examined to find the number of times each code was identified by the participants [33].

The first and the last author categorized all possible OPIs into the five TCOP levels individually, followed by a discussion that led to a uniformed categorization called “author's vote.” To study the variation of the categorization process, the additional four OT lecturers contributed with their own ratings and the “majority vote” consisted of an agreement of three or more raters. For both votes, frequencies and percentages were calculated for each of the five levels of the TCOP within the entire population and the three occupational areas (S, P, and L). Homogeneity of the TCOP levels was tested across occupational areas in both the authors vote and the majority vote with Fisher's exact test. To evaluate interrater agreement, we calculated Fleiss' kappa statistic and determined the interrater agreement by the following classification: ≤0.20 poor, 0.21-0.40 fair, 0.41-0.60 moderate, 0.61-0.80 good, and 0.81-1.00 very good (34, p. 404). Stata version 16 was used [34], and for all statistical tests, a *p* value < 0.05 was considered statistically significant.

## 3. Results

### 3.1. Study Population

141 participants (*n* = 57 males, 40%) were eligible and met the inclusion criteria, and 112 participants consented (*n* = 49 males, 44%) with a mean age of 65.2 years (range 16-96 years). About half of the participants (*n* = 63, 56%) were recruited from the regional hospital and the rest (*n* = 49, 44%) from the rehabilitation centre. The entire study population is displayed in population groups with gender and age in Table 2.

### 3.2. The Construct of the COPM Related to the CMOP-E

**Figure 1:**
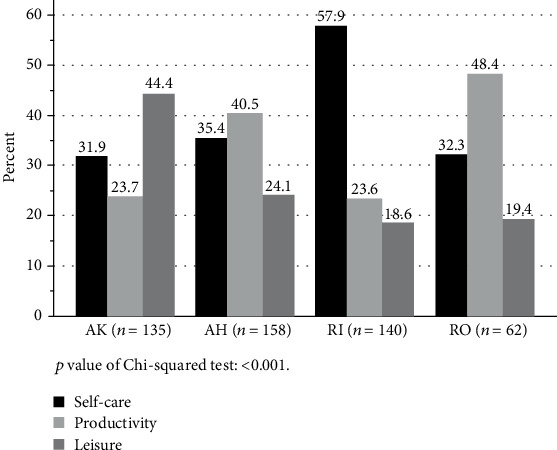
Distribution of the identified OPIs in the four population groups. AK: regional clients, knee replacement; AH: regional clients, hand surgery; RI: community clients from a rehabilitation centre, inpatient department; RO: community clients from a rehabilitation centre, outpatient department.

The entire study population prioritized 495 OPIs, divided into self-care (*n* = 200, 40%), productivity (*n* = 159, 32%), and leisure (*n* = 136, 28%). As shown in Figure 1, the distribution of the three OPI areas were significantly different within each of the four population groups (AK, AH, RO, and RI) (*χ*^2^ (df) = 50.06, *p* < 0.001) [6]. In the two regional groups, the OPIs were equally divided into the three OPI areas, with most leisure OPIs (44.4%) identified in population AK and most productive OPIs (40.5%) in population AH. The OPIs identified by the RO population were also quite equally divided, with most productive OPIs (48.4%) prioritized. A larger diversification was seen in the RI population, who identified more than half of the OPIs within self-care (57.9%) with less than 25% of the OPIs within productive and leisure OPIs each.

Some OPIs were mentioned by several clients which led to the identification of a total of 224 different OPIs.  Table 3 presents examples of the OPI coding in the different areas.

The frequencies of the OPIs with at least ten incidents can be seen in Table 4. The three most frequently mentioned OPIs all belonged to the self-care area, with “taking a bath/shower (SA1)” being mentioned most times (*n* = 37 times, 7.5%), followed by “walking (SB1)” (on stairs up to the 2^nd^ floor, toilet, and bedrooms) (*n* = 32, 6%), and “dressing (SA7)” (*n* = 24, 5%).

### 3.3. The Construct of the COPM Related to the TCOP

Based on the 224 different OPIs, the “authors' vote” within each occupational performance area is displayed in Table 5 and led to the following classification: 144 (64.3%) OPIs were classified in the occupation/activity levels, 54 (24.1%) OPIs were identified in the task level, whereas 26 (11.6%) OPIs were classified in the two lowest levels, action and voluntary movements. A significant difference was found in the OPIs' distribution in the levels of TCOP (*χ*^2^ (df) = 108.3, *p* < 0.001) [8].

**Figure 2:**
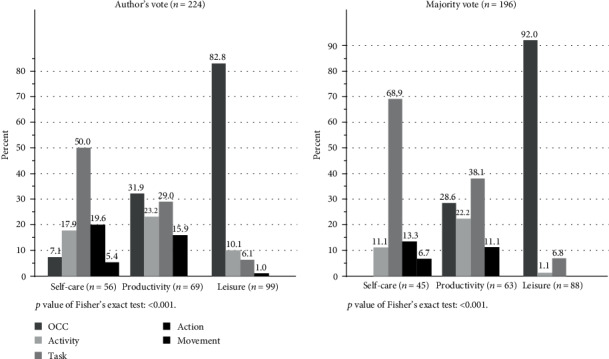
The classification of OPIs on TCOP levels, authors' vote, and majority vote.

For each item (OPI, *n* = 224), we looked at the maximum number of rater agreeing on a categorization and made a “majority vote” based on a minimum of three rater's agreements (*n* = 28/224), since at least three raters needed to agree for a majority. Of the 28 OPIs where less than three raters agreed, 11 were within self-care, eight within productivity, and nine within leisure. In most cases, the raters classified the OPIs as either tasks, activities, or occupations. However, in three cases, four levels were used as actions which included staying dry at night (SA27), swim (LB13), and go Nordic (LB25). The “majority vote” is shown in Figure 2, and the differences between the “authors' vote” and the “majority vote” are depictured. Significant difference (*p* = <0.01) in the TCOP level distribution were found across the different main occupational areas (S, P, and L). To investigate variation, we estimated the interrater agreement by the Scott/Fleiss' kappa statistic and found fair agreement (0.30 with 95% confidence interval 0.26-0.34) [33].

## 4. Discussion

This study examined the content validity of the COPM-DK in two parts. First, we examined how the Danish clients identified the OPIs, reflecting the construct of occupational performance as conceptualized by the CMOP-E, and second, how these OPIs related to the levels of the TCOP as perceived by six Danish OTs. The study showed that Danish clients identified very different OPIs within the three areas of occupational performance and that the OPIs were classified in all the five levels described in the TCOP. Since the participating raters did not agree on how to classify the OPIs and the interrater agreement was only fair, the description of how the OPIs could be classified within of the levels of the TCOP was based on the “authors' vote.” According to this, 35.7% of the OPIs were classified to the lower levels, i.e., as tasks, actions, or voluntary movements.

### 4.1. The OPIs of Danish Clients Related to the CMOP-E

The total population of Danish clients identified OPIs that were divided almost evenly within the three areas of occupations, whereas the division of OPIs within each of the four population groups differed significantly. The participants identifying the most leisure OPIs were the population who had knee replacement (AK), while the populations who had hand surgery (AH) or received rehabilitation as outpatients in the community (RO) mostly prioritized productive OPIs. The largest diversity in the prioritized OPIs was seen in the population of inpatients in the community (RI) with more than half of the OPIs within self-care. This can be interpreted as the COPM's client centredness [4], as it reflects the COPM's ability to identify how clients within a specific age who suffer from certain diseases or injuries perceive their everyday life. It makes sense that only one OPI was identified in group PC (attending school and play) in this study population having a mean age of 65.2 years. It is also understandable that clients with a mean age of 67 years, i.e., past the retirement age in Denmark, who had knee replacement (AK), generally identified leisure-related OPIs, while clients mostly being in the working age (AH and RO) identified most productive OPIs. It also seems logical that clients with a mean age of 77 years who are inpatients at a rehabilitation centre, identified most OPIs within self-care and fewer within productivity and leisure. Similar distributions of OPIs have been identified in other studies [35, 36]. Even if most studies on the COPM examine certain groups of clients, some studies have examined a diverse group of clients and have shown similar differences in the distribution of OPIs with variations related to the different settings, conditions, and ages [25, 37, 38].

The clients identified most of the OPIs in self-care (*n* = 200, 40%), as seen in other studies [36, 37]. Because the COPM is known to help clients express what they really find distressing in their present lives, this finding makes sense, as many adults may express frustration and maybe humiliation with not being able to perform the self-care activities that they learned in childhood. As such, it can be interpreted as an example of the COPM's ability to help clients identify issues with the occupations that really matter to them in their current situation [2, 4, 39]. However, this could also be interpreted as the OTs' preference for the self-care area, as OTs often takes responsibility for training the clients' self-care abilities [5, 11, 25, 37]. Bearing this in mind, OTs must listen carefully to the clients to enable that the clients express what *they* really want to change and not what they expect the OTs to handle.

### 4.2. The OPIs of Danish Clients Related to the TCOP

Our analyses classified 54 (24.1%) OPIs in the task level, and 26 (11.6%) OPIs in the two lowest levels of actions and voluntary movements. Although the authors of the COPM have emphasize that “while it is recognized that attention to underlying skills in performance components is essential to OT, these components support, rather than define, occupational performance. As such, they do not constitute a focus of the COPM” (1, p. 3), the distinction of what to identify when using the COPM has not been subjected to many discussions in the literature. Perhaps this has contributed to the inattention to or unawareness of this issue. However, the phenomenon that clients identify issues on all levels, including tasks or actions, have previously been seen in studies of the COPM [40–43]. In a study of Poulsen and Hansen, a vast number of the identified OPIs were classified in the lower levels of the TCOP [41]. Similarly, in their study, Nieuwenhuizen et al. found that a little over 30 percent of the prioritized OPIs could be classified as problems at the level of body and mental functions of the International Classification of Functioning (ICF) [44], thus comparable to the lower levels of the TCOP. This apparent inconsistency in what to assess or identify with the COPM affects its validity, as it may reflect a misunderstanding of the measurement's construct. This lack of precision may have been engendered by the COPM manual itself, as examples of lower level issues were suggested as OPIs in the former Appendix A of the manual [25, 26]. Therefore, in February 2019, the authors removed this Appendix from the revised 5^th^ edition of the COPM manual and have rewritten the examples of OPIs more in line with the top levels of the TCOP [45]. Even so, most COPM users have learnt to use the measurement based on former editions of the COPM manual, and it may take some time to unlearn how the COPM has been used [5, 25].

The inconsistency could also arise from a lack of precision in the way the COPM is administered. Studies of the utility of the COPM have revealed that some OTs use the COPM to identify what constitutes the OPIs to enable setting exact goals for rehabilitation. According to the Canadian models of OT intervention [1, 16], identifying OPIs with the COPM, analysing what constitutes the OPIs, and formulating goals for rehabilitation are different steps in the OT intervention process. The COPM is known as an effective tool for goal setting. However, the risk of deflecting the content of the COPM to the lower levels of the TCOP increases, if these steps are done together with the COPM interview in one session. Therefore, to include the process of analysis and goal setting in a COPM interview should be handled with the utmost caution. If not, it risks challenging the construct of the measurement. However, these elements should surely be included as important parts in an OT rehabilitation process.

The highest number of OPIs classified in the lower levels of the TCOP was included in the area of self-care. This might be a result of how the OTs administered the COPM, as some OTs have noted that the more precise the OPIs are formulated, the easier the clients find the rescoring process [11, 12, 41]. Therefore, some OTs may steer the interview so the clients identify OPIs that are more concrete formulated, i.e., on lower levels of the TCOP [11, 12, 46]. If, however, the COPM's content truly is *occupational* performance, it is important not to steer the clients to formulate OPIs on the levels of tasks and actions. If the clients volunteer these issues because they find them important, the OTs should acknowledge their wishes and go with that. Nevertheless, self-care tasks should not be identified as a result of a well-meaning OT with a preference for solving these basic problems [47]. In order to claim that the COPM is a measure that validly represents the entity of occupational performance, the construct must be understood and carefully defined with selected items that represent that definition, while considering that specific wishes from clients may contradict the description.

Finally, our study revealed a considerable difference in how skilled OTs classified the identified OPIs within the levels described by the TCOP, with a kappa coefficient on 0.30 (a fair agreement). This might by imposed by a lack of precision in the description of the levels included in the TCOP—and the ongoing discussions on the content of the concepts of occupations and occupational performance within OT also illustrates this lack of precision [18, 48, 49]. However, enhancing the precision of the TCOP levels is beyond this study. More studies are needed in order to establish a more precise taxonomy on the content of the concept of occupational performance.

Since measurement scores collected in clinical practice and research provide the foundation on which the results of a rehabilitation process rest, its success or failure depend upon the quality of these scores. Thus, to ensure the clinimetric standards of a measurement, the rater agreement is important [50]. Despite the fact that clinimetric studies on the COPM have included courses in administrating the COPM as rater training [25, 40, 44, 51], this is not a formal requirement [26, 45]. Users are only required to read the COPM manual and to be familiar with the concepts on which it is constructively derived for administering and scoring [45]. Our study, as well as other studies, has shown that this might not be a straightforward task [5, 11, 15]. Danish OTs complete their authorization as skilled OTs in an academic programme which includes learning about the concepts of occupations and occupational performance, and similar knowledge cannot be expected to be included in the education of other health professionals. Although educated with an occupational perspective and having received an introductory course prior to administering the COPM in this study, OTs develop an individualized paradigm related to their settings and beliefs over time [52]. This might have imposed a variety in the understanding, administering, and interpretation of the COPM, leading to the differences seen in the identification as well as the interpretation and classification of the identified OPIs.

Since this study have revealed issues that need special attention when administering the COPM interview, such as intimate knowledge of what to identify in the interview, e.g., what an OPI is, we would suggest that an introductory course should be mandatory. We are aware that this is not the recommendation of the authors; however, in order to perform the COPM validly, it could seem necessary. Furthermore ongoing support is recommendable [4, 25, 45, 53] since studies have shown that structured rater training has a positive effect on OT scoring performance when using other measurements translated to Danish [54].

### 4.3. Strengths and Limitations of the Study

A population of 112 participants from various settings and with a broad range of medical issues was included to examine the content validity of the COPM, identifying 224 different OPIs. The participants represented a wide spectrum of OT clients seen in Danish OT practice of physical rehabilitation. Despite our thorough efforts, the external validity might be limited, due to only including one hospital and one rehabilitation centre in the Capital Region of Denmark. Including clients from other settings or other regions of Denmark could have increased the extern validity.

Content validity is best measured by experts, who have the ability to understand the specialized concepts of the measurement examined [55]. However, as opposed to a self-administered questionnaire, the COPM is administered as a semistructured interview. The COPM therefore includes two different expert panels: the clients, who deliver the items (the OPIs) within the concept, and the OTs, who understands the concept on which the COPM is based. Therefore, this study included a purposeful sampling of fourteen OTs as an expert panel, i.e., the first author and last author, the eight OTs from the two settings, and the four OT lectures. The OTs administered the COPM interviews with the participating clients based on an introductory course to ensure a range of clinical expertise. Although a common interview style was sought through the courses held by the first author in both settings, every OT has his or her own style of interviewing as well as their own beliefs and values. This may have enhanced the variation of the outcome. However, as the COPM is meant to be generic and used preferably by OTs and also by other health care professionals, the data obtained by the COPM should not vary too much despite individual interview styles.

The other groups of experts were the included OT lecturers. They were involved as experts to conceptualize the OPIs in the classifying process with the TCOP. Classifying the OPIs within the TCOP as an aspect of content validity examination has not been exercised previously, but similar methods have been used in validation studies when linking a measurement to the ICF [41]. Although the included lecturers were familiar with the COPM and the TCOP, they were challenged when establishing meaningful concepts and consensus in the process. This was partly due to the coding process by the first author, as OPIs that belonged to the same topic were put together under the same code. For instance, the code SA2 included “to put on pants, jeans, underpants, skirt.” This could be interpreted as putting on all clothes, i.e., dressing and thus an occupation, whereas it contained the four different tasks of putting on each item on its own. Similarly, the code LC11 “attend concerts, standing” both include participating in a concert, i.e., an occupation, and the part of “standing at the concert” as an action.

The experience of an occupation is individual, and looking from the outside, we cannot know whether an occupation is performed regularly, considered to be complex, and held a meaning to the client. Thus, classifying OPIs based on notes made by the interviewing OTs that lack this important information makes the classification uncertain, and it must be considered with caution. It would probably have increased the validation if notes were made by the OTs who included this information to secure that the clients' meaning of the OPIs were correctly interpreted. The included lectures also found the classification challenging due to the lack of precision in the levels of the TCOP. Before a classification procedure, a practice period and an established consensus of the classification levels of the TCOP would have been preferable. More studies on the content of the TCOP as well as the content of the concept of occupational performance are thus warranted.

It is reasonable and expected that an instrument validation is more difficult for clients than for OTs or health professionals, as clients have limited knowledge of the construct [56, 57]. However, a measurement must be clear to all respondents, even if they do not understand the underlying concepts. It seems that the COPM does inflict some uncertainties as to what it measures. Even if the COPM covered a broad spectrum of OPIs in the areas of self-care, productivity, and leisure, we found that some OPIs were in fact not *occupations* but could be classified as lower levels and apparently not within the concept of the COPM. However, since clients can nominate any OPI that is important to them, the identified OPIs might be within the client-centred aspect of the COPM.

## 5. Conclusion

This study supports the content validity of the COPM-DK for clients receiving rehabilitation in both the regional and municipal settings. The data obtained were diverse, interpreted as a result of the COPM's ability to facilitate a client-centred approach and obtain OPIs that represent the clients' engagement and values. As more than one-third of the identified OPIs were classified to levels below the occupation and activity levels in the TCOP, it questions if the data obtained with the COPM represent the construct of the measurement. Therefore, to secure that OPIs identified with the COPM reflect the content of *occupational performance*, caution must be taken to secure that the COPM interview remains on the higher levels of the TCOP, while keeping up the clients' right to nominate any OPI that is important to them. In conclusion, given attention to the content of the COPM, the COPM seems to be a useful interview-based measurement to ensure a client-centred rehabilitation.

## Figures and Tables

**Table 1:** Description of the five levels of the Taxonomic Code of Occupational Performance (TCOP) (17, p. 19).

Levels of complexity	Definition
1. Occupation	The overarching concept of doing. Performed with some consistency or because it gives meaning and value to life, e.g., having a birthday dinner or getting a job
2. Activity	The doings within an occupation. Have specific outcomes, e.g., planning and preparing food for the dinner or writing a job application
3. Task	A performance with a lesser and more specific outcome, e.g., preparing a cucumber salad or printing the job application
4. Action	Voluntary movements or mental processes that form a recognizable and purposeful pattern, e.g., holding on and cutting cucumbers for the salad with a knife or grabbing the paper from the printer
5. Voluntary movements or mental processes	The basic body functions (i.e., joint motions or cognitive abilities) needed to perform tasks, e.g., flexing the fingers around the knife and knowing the right sizes of cucumbers needed for this particulate salad and flexing the fingers to grasp the paper and pay attention to the print when it is ready.

**Table 2:** Sample characteristics of the participants (*n* = 112).

	Regional participants (*n* = 63, 56%)		Community participants (*n* = 49, 44%)	
	AK (*n* = 31, 50%)	AH (*n* = 32, 50%)	RI (*n* = 34, 69%)	RO (*n* = 15, 31%)
Gender (m/f)	16/15	16/16	11/23	6/9
Age (mean yrs (sd))	67 (8.3)	56 (18.5)	77 (6.7)	67 (15.1)
Age, range (yrs)	Min 48, max 81	Min 16, max 96	Min 64, max 90	Min 31, max 83

AK: regional clients from a hospital, knee replacement; AH: regional clients from a hospital, hand surgery; RI: community clients from a rehabilitation centre, inpatient department; RO: community clients from a rehabilitation centre, outpatient department; yrs: years; sd: standard deviation.

**Table 3:** Examples of the coding of the prioritized OPIs (*n* = 224).

Self-care (*n* = 56)					
SA, personal care (*n* = 29)		SB, functional mobility (*n* = 18)		SC, community management (*n* = 9)	
Shower, take a bath	SA1	Walk (on stairs) up to the 2^nd^ floor, toilet, bedroom	SB1	Operate pc, write, spell	SC1
Put on pants, jeans, underpants, skirt	SA2	Get in/out of the car	SB2	Manage banking	SC2
Use the toilet—get on, dry after, take off/put on clothes, press rinse-button	SA3	Get in/out of the house	SB3	Talk on the phone	SC3
Put on socks	SA4	Walk to/in town/walk outside	SB4	Contact authorities	SC4
Put on/remove support socks, leg rail	SA5	Move freely, walk around in one's own home	SB5	Get to the doctor/dentist	SC5
Cut nails on toes	SA6	Move freely	SB6		

Productivity (n =69)					
PA, paid or unpaid work (*n* = 25)		PB, household management (*n* = 43)		PC, school and play (*n* = 1)	
Work	PA1	Shopping	PB1	Attend studies	PC1
Managing a café	PA2	Doing the dishes	PB2		
Looking after children, grandchildren, lifting up, changing diapers	PA3	Vacuuming	PB3		
Looking after spouse	PA4	Maintain cottage, colonial garden	PB4		
Getting to and around work	PA5	Keep the garden	PB5		
Attending meetings, standing	PA6	Cleaning	PB6		

Leisure (*n* = 99)					
LA, quiet recreation (*n* = 32)		LB, active recreation (*n* = 39)		LC, socialization (*n* = 28)	
Managing interior design	LA1	Take long walks	LB1	Visit friends & family, living with stairs	LC1
Making flower arrangements	LA2	Bike on exercise bike	LB2	Playing, doing activities (on the beach) with children, grandchildren	LC2
Paint pictures (e.g., of flowers), outside.	LA3	Take bike rides	LB3	Meet, be on trips with friends	LC3
Go for a morning walk and inspect the garden	LA4	Jog, run	LB4	Participate in activities (e.g. coffee club) with family & friends	LC4
Join a knitting club	LA5	Play tennis	LB5	Participate in folk school	LC5
Building model aircrafts	LA6	Walk the dog	LB6	Have guests	LC6

**Table 4:** The top ten frequencies of the identified OPIs (*n* = 495).

	OPI code	Frequency (*n*)	Percent (%)
Shower, taking a bath	SA1	37	7.47
Walking (on stairs) up to the 2nd floor, toilet, bedroom	SB1	32	6.46
Dressing	SA7	24	4.85
Working	PA1	23	4.65
Cooking while standing	PB9	22	4.44
Biking	SB4	16	3.23
Cleaning	PB6	15	3.03
Eating with cutlery, utensils	SA14	15	3.03
Doing fitness, workout	LB7	14	2.83
Keeping the garden	PB5	14	2.83
Taking long walks	LB1	13	2.63
Driving a car	SB5	11	2.22
Shopping	PB1	10	2.02

SA: self-care, personal care; SB: self-care, mobility; PA: productivity, work; PB: productivity, household; LB: leisure, active recreation.

**Table 5:** Examples of OPIs classified within the TCOP (*n* = 224).

	*Self-care*	*Productivity*	*Leisure*
Levels of occupation (1) and activity (2)	e.g., taking a shower, sleeping, having sex, putting on clothes, personal hygiene, styling (hair, makeup), driving a car, walk in the city, taking public transportation, handling finances, dealing with authorities and health care professionals	e.g., managing paid or unpaid work, taking care of children, grandchildren, parents or pets, managing housework, i.e., cleaning, cooking, shopping, maintain house, garden, boat, studying	e.g., participating in hobbies, e.g., painting, needlework, managing interior design, paint pictures (e.g., of flowers), playing music instruments or singing, going on holidays, socializing with friends and family, doing sports, go to the cinema, and the like
OPIs (*n*) %	Level 1 (*n* = 4, 7.1%) Level 2 (*n* = 10, 17.9%)	Level 1 (*n* = 22, 31.9%) Level 2 (*n* = 16, 23.2%)	Level 1 (*n* = 82, 82.8%) Level 2 (*n* = 10, 10.1%)
Level of task (3)	e.g., put on pants, jeans, underpants, skirt, socks, shoes; cutting nails, combing hair, brushing teeth, getting in-out of cars, getting up and down from chairs, climbing stairs, opening/closing doors, talking in phones, handling the computer, standing	e.g., write by hand, use laptop, bring and fetch children, use mobile, cell phone, filling, emptying washing machine/dishwasher, change tires, repair car, polish windows, get ready for window cleaning, mowing the lawn, hang clothes up/on hangers, wash the floor, getting to and around on work	e.g., writing, using PC/social media/mobile phone, travel getting in-out of planes, go around in airports and cities
OPIs (*n*) %	Level 3 (*n* = 28, 50.0%)	Level 3 (*n* = 20, 29.0%)	Level 3 (*n* = 6, 6.1%)
Level of action (4), movement or mental abilities (5)	e.g., zip the zippers, thigh buttons, do fasteners, keep, stay dry at night, speak, get up and down from a chair, getting in/out of bed, get up from the floor, use fine motor skills	e.g., lift heavy things, carry laundry, make repairs from or put things in place from ladder, remove things from the floor for cleaning, tie knots, opening containers	e.g., write in hand, standing while attending activities e.g. concerts, visits, lectures, stretch and hand things over the table
OPIs (*n*) %	Level 4 (*n* = 11, 19.6%) Level 5 (*n* = 3, 5.4%)	Level 4 (*n* = 11, 15.9%) Level 5 (*n* = 0, 0.0%)	Level 4 (*n* = 1, 1.0%) Level 5 (*n* = 0, 0.0%)

Level 1: occupations. Level 2: activities. Level 3: tasks. Level 4: actions. Level 5: movements of mental abilities.

## Data Availability

Access to data is restricted. Complying with European data protection rules, the Copenhagen University College approved the data processing activities regarding this project and registered the project (project number 18-025). Restricting access is due to legal and ethical concerns. We are according to the Danish data protection legislation not allowed to submit the data or give access to the data used for the analyses.
